# A Composite Risk Score Based on VI-RADS, Tumor Contact Length, and CYFRA 21-1 for Prognostic Stratification in Bladder Cancer

**DOI:** 10.3390/diagnostics15232968

**Published:** 2025-11-22

**Authors:** Shunsuke Ikuma, Jun Akatsuka, Godai Kaneko, Hayato Takeda, Yuki Endo, Go Kimura, Yukihiro Kondo

**Affiliations:** Department of Urology, Nippon Medical School, 1-1-5 Sendagi, Bunkyo-ku, Tokyo 113-8603, Japany-endo1@nms.ac.jp (Y.E.); gokimura@nms.ac.jp (G.K.);

**Keywords:** bladder cancer, tumor contact length, cytokeratin 19 fragment, magnetic resonance imaging, reporting and data system, Vesical Imaging-Reporting and Data System

## Abstract

**Background/Objectives**: The Vesical Imaging-Reporting and Data System (VI-RADS) provides high diagnostic accuracy for muscle-invasive bladder cancer; however, its prognostic value remains limited. We propose serum cytokeratin 19 fragment (CYFRA 21-1) and tumor contact length (TCL) as complementary prognostic factors. We aimed to construct a composite risk score integrating VI-RADS, CYFRA 21-1, and TCL for prognostic stratification. **Methods**: We retrospectively analyzed data from 101 patients with bladder cancer (BC) who underwent transurethral resection of bladder tumor (TURBT), magnetic resonance imaging, and postoperative serum CYFRA 21-1 measurement. For each factor, cut-off values were determined using receiver operating characteristic (ROC) analysis; meeting each threshold contributed one point (score range, 0–3). Overall survival (OS) was assessed using Kaplan–Meier and Cox regression analyses. **Results**: ROC analysis identified cut-offs of VI-RADS ≥ 3 (area under the curve [AUC] 0.779), TCL ≥ 40 mm (AUC 0.817), and CYFRA 21-1 ≥ 2.1 ng/mL (AUC 0.875). Based on these, patients were stratified into low- (0–1, *n* = 81), intermediate- (2, *n* = 12), and high-risk (3, *n* = 8) groups with 3-year OS rates of 95.1%, 75.0%, and 25.0%, respectively (*p* < 0.001). In univariate Cox regression, all factors significantly predicted poor OS: VI-RADS ≥ 3 (hazard ratio [HR], 6.51; *p* = 0.015), TCL ≥ 40 mm (HR, 8.36; *p* < 0.001), and CYFRA 21-1 ≥ 2.1 ng/mL (HR, 14.02; *p* < 0.001). In multivariate analysis, only CYFRA 21-1 remained independently significant (HR, 11.80; *p* < 0.001). **Conclusions**: A composite risk score combining VI-RADS, TCL, and CYFRA 21-1 effectively stratified patients with BC into distinct groups using minimally invasive, peri-TURBT assessments. Prospective multicenter validation is warranted.

## 1. Introduction

The Vesical Imaging-Reporting and Data System (VI-RADS) has become an established, standardized framework for the multiparametric magnetic resonance imaging (MRI) evaluation of bladder cancer (BC), with proven diagnostic performance in distinguishing between muscle-invasive bladder cancer (MIBC) and non-muscle-invasive bladder cancer (NMIBC) [1]. VI-RADS has demonstrated high diagnostic accuracy for MIBC in meta-analyses [2]. In recent years, the utility of VI-RADS has extended beyond predicting MIBC; efforts have been made to expand its application to prognostic prediction by incorporating complementary factors.

Recently, serum tumor markers have attracted attention as minimally invasive tools for the risk stratification of solid tumors including BC. These markers are considered useful adjuncts to imaging diagnostics, as they may reflect tumor biology, tumor burden, and treatment response. Several reviews have emphasized the clinical importance of serum tumor markers in MIBC, highlighting their potential roles in diagnosis, prognosis, and disease monitoring [3]. Cytokeratin 19 fragment (CYFRA 21-1) is one such marker. As a soluble fragment of cytokeratin 19—a protein expressed in epithelial cells and associated with differentiation—CYFRA 21-1 can be measured in serum and is frequently elevated in a range of solid tumors including BC [4,5,6]. Combining VI-RADS with serum CYFRA 21-1 has recently been reported to facilitate the stratification of patients with BC into groups with poorer prognosis [7].

The morphology of the tumor base provides important information for predicting muscle invasion in BC. The presence of a broad-based tumor is associated with an increased risk of muscle invasion. Tumor contact length (TCL) refers to the extent of contact between the tumor and the bladder wall. Ozden et al. demonstrated that a longer TCL, as assessed using ultrasonography, was associated with an increased likelihood of MIBC [8]. Local diagnosis of BC is increasingly performed using MRI, particularly with the widespread application of VI-RADS. Several studies have reported that measuring TCL on MRI and combining it with VI-RADS assessment improves the diagnostic accuracy for MIBC [9,10,11]. TCL closely reflects tumor size, which is a recognized risk factor for tumor recurrence and progression [12]. Moreover, adding TCL as a complementary factor to VI-RADS in diagnosing MIBC may provide additional value for prognostic prediction.

This study aimed to evaluate whether the utility of VI-RADS could be expanded to include prognostic prediction. Traditional prognostic models, such as the EORTC risk tables, were developed before the widespread use of multiparametric MRI and the growing clinical relevance of serum biomarkers such as CYFRA 21-1 [12]. Updating prognostic models by incorporating imaging-based parameters and serum biomarkers may therefore provide more accurate and individualized risk assessment in the current era. To this end, we developed a composite risk score integrating VI-RADS with serum CYFRA 21-1 and TCL as complementary factors and assessed its ability to improve prognostic stratification in patients with BC. Validation of this risk score could enable patient stratification at an early stage in the clinical pathway during the peri-TURBT period. Risk stratification tools are essential in BC as they can identify high-risk patients who may benefit from early aggressive interventions while sparing low-risk patients from overtreatment.

## 2. Materials and Methods

### 2.1. Study Population

This single-center, retrospective study included patients who underwent TURBT and were pathologically diagnosed with urothelial carcinoma, and who had both preoperative MRI and postoperative CYFRA 21-1 measurement between August 2021 and July 2023. Inclusion criteria were as follows: (1) patients who underwent TURBT during the study period; (2) pathologically confirmed urothelial carcinoma; (3) preoperative MRI available for assessment of VI-RADS and TCL; and (4) postoperative CYFRA 21-1 measurement performed. Exclusion criteria were as follows: (1) lack of preoperative MRI; and (2) MRI image quality insufficient for reliable VI-RADS or TCL assessment. Eligible patients were identified through a review of institutional electronic medical records. Four patients were excluded because poor MRI quality prevented the assessment of VI-RADS and morphological measurements. The study protocol was approved by the Institutional Review Board (IRB) of the Nippon Medical School Hospital (approval no. F-2023-049, approved on 5 July 2023) and adhered to the principles of the Declaration of Helsinki. Given the retrospective design, the requirement for written informed consent was waived. However, an opt-out option was provided to all patients through the IRB website.

### 2.2. Image Acquisition

**Figure 1:**
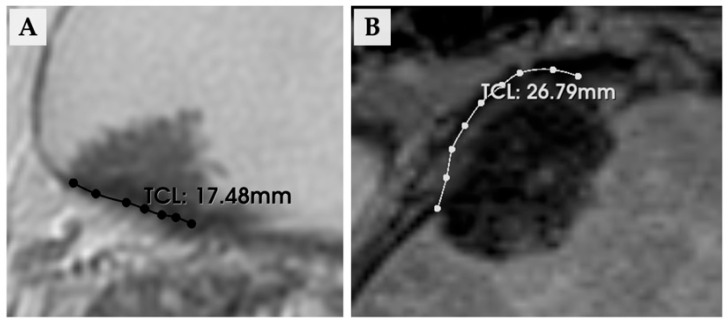
Representative examples of tumor contact length (TCL) measurement on axial T2-weighted magnetic resonance images. (**A**) A 64-year-old man with a TCL of 17.48 mm; pathological diagnosis was pTa. (**B**) A 69-year-old woman with a TCL of 26.79 mm; pathological diagnosis was pT2. Abbreviations: TCL, tumor contact length.

The overall VI-RADS score (range, 1–5) was determined through an integrated assessment of T2-weighted imaging (T2WI), diffusion-weighted imaging (DWI), and dynamic contrast-enhanced (DCE) MRI, following the original recommendations of Panebianco et al. [1]. When DCE-MRI was unavailable, biparametric MRI (T2WI and DWI) was performed, as described by Noh et al. [13]. Any discrepancies between the two evaluators were resolved through consensus discussions, with a radiologist providing illustrative cases to ensure consistent grading. To minimize interpretation bias, both MRI readers were blinded to all outcome information including pathological results and serum CYFRA 21-1 levels. VI-RADS evaluation was conducted by two urologists: one with more than 10 years of clinical experience including five bladder MRI readings, and another with over 5 years of clinical experience and three bladder MRI evaluations. TCL was defined as the longest curvilinear distance of attachment between the bladder wall and the tumor on axial T2-weighted MR images. The axial slice demonstrating the most extensive tumor–wall interface was selected, and the contour of the contact area was carefully traced along the boundary between the lesion and the bladder wall. TCL values were recorded in millimeters (mm). In addition, we specified the measurement software used in this study. TCL was measured using 3D Slicer (version 5.8.1; NA-MIC, Boston, MA, USA), which enabled precise curvilinear measurement along the tumor–bladder interface on T2-weighted images. To facilitate understanding, illustrative examples of the TCL measurement method are provided in Figure 1.

### 2.3. Techniques and Procedures in TURBT

During TURBT, the surgeons resected the entire visible tumor burden along with two deeper layers from the index region to obtain representative samples. The collected tissues were preserved in 10% formalin and subjected to pathological evaluation. Muscularis propria invasion was assessed by an expert uropathologist with more than two decades of professional experience.

### 2.4. Serum Collection

Serum CYFRA 21-1 levels were measured using an electrochemiluminescence immunoassay (Elecsys CYFRA 21-1, Roche Diagnostics, Mannheim, Germany). Blood sampling was performed after TURBT to minimize the potential confounding effect of tumor size on circulating levels and reflect systemic disease activity. Serum CYFRA 21-1 was measured postoperatively, with a median sampling time of 1 day (interquartile range [IQR], 1–1 day) and a mean of 3.5 days (standard deviation, 13.9). More than 90% of patients had samples collected within 2 days of TURBT.

### 2.5. Study Workflow and Statistical Methods

First, the VI-RADS scores were assessed. Next, receiver operating characteristic (ROC) curves were constructed using the VI-RADS, TCL, and CYFRA 21-1 values to predict 1-year survival. Cut-off values were determined using Youden’s index. For each factor, values above the cut-off were assigned 1 point, yielding a maximum total score of 3 points. Patients were categorized into three risk groups: low risk (0–1 point), intermediate risk (2 points), and high risk (3 points). Clinicopathological characteristics were summarized across the three groups. Kaplan–Meier survival analysis was performed to compare survival differences among the risk groups, and a Cox proportional hazards regression analysis was conducted to evaluate the prognostic impact of individual clinicopathological factors. A post hoc power analysis based on the log-rank test was additionally performed to confirm that the study had sufficient statistical power to detect the observed survival differences.

### 2.6. Outcome of Treatment

The primary outcome of this study was OS, defined as the time from TURBT to death, which was compared across the risk groups classified by the composite risk score. Patients who were alive at the last clinical follow-up were censored at that date. The secondary outcome was subgroup OS, evaluated separately in the treated subgroup—which included patients who underwent systemic chemotherapy or radical cystectomy—and the untreated subgroup, consisting of patients who received neither systemic chemotherapy nor radical cystectomy. This secondary analysis was performed to determine whether the composite risk score could stratify prognosis consistently across different treatment contexts.

### 2.7. Statistical Analyses

We evaluated the diagnostic performance of VI-RADS for detecting MIBC. Inter-reader agreement was assessed using the weighted kappa coefficient. The cut-off values for TCL and CYFRA 21-1 were determined using Youden’s index. Cox regression analysis was used to estimate hazard ratios (HRs) and 95% confidence intervals (CIs) for each variable. Continuous variables were first tested for normality using the Shapiro–Wilk test. Normally distributed variables were compared among the three risk groups using one-way ANOVA, whereas non-normally distributed variables were analyzed using the Kruskal–Wallis test. Categorical variables were compared using the chi-square test or Fisher’s exact test, as appropriate. All statistical analyses were performed using SPSS version 29.0 (SPSS Inc., Chicago, IL, USA) and GraphPad Prism version 10.0 (GraphPad Software, San Diego, CA, USA). The intraclass correlation coefficient (ICC) for interobserver agreement of TCL measurement was also calculated using SPSS to evaluate measurement reproducibility. A post hoc power analysis based on the log-rank test was additionally performed using MedCalc (MedCalc Software Ltd., Ostend, Belgium) to confirm that the study had sufficient statistical power to detect the observed survival difference among the risk groups. A *p*-value < 0.05 was considered statistically significant.

## 3. Results

**Figure 2:**
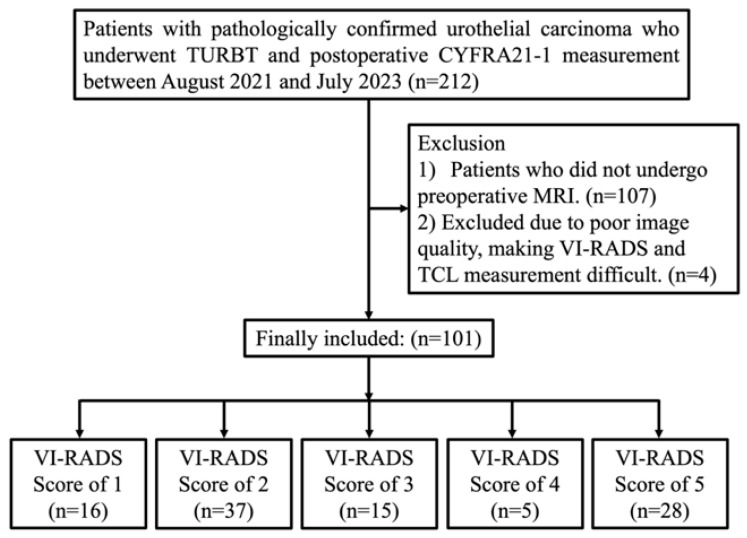
Patient selection flowchart for final analysis. Abbreviations: TURBT, transurethral resection of bladder tumor; MRI, magnetic resonance imaging; CYFRA 21-1, cytokeratin 19 fragment; TCL, tumor contact length; VI-RADS, Vesical Imaging-Reporting and Data System.

A total of 212 patients with pathologically confirmed urothelial carcinoma who underwent TURBT and had postoperative CYFRA 21-1 measurements between August 2021 and July 2023 were initially identified. Among them, 107 patients who did not undergo preoperative MRI and 4 patients whose MRI images were of insufficient quality for reliable VI-RADS or TCL assessment were excluded. Consequently, 101 patients were included in the final analysis and categorized according to their VI-RADS scores, as shown in Figure 2.

In this study, the median follow-up duration was 26.6 months, during which 13 patients (12.8%) died. A VI-RADS cut-off score of 4 showed an area under the curve (AUC) of 0.89, with 87.1% sensitivity, 91.4% specificity, 90.1% accuracy, 81.8% positive predictive value, and 94.1% negative predictive value. A VI-RADS score of 4 demonstrated better diagnostic performance than other cut-off scores. The agreement between the two readers was substantial, with kappa statistics of 0.84 (95% CI, 0.77–0.91) for the T2WI score, 0.87 (95% CI, 0.80–0.93) for the DWI score, 0.82 (95% CI, 0.73–0.91) for the DCE score, and 0.85 (95% CI, 0.79–0.92) for the overall VI-RADS score. Excellent agreement was observed between the readers in the assessment of VI-RADS scores. The interobserver agreement for TCL measurement was also excellent, with an ICC of 0.91 (95% CI: 0.87–0.94, *p* < 0.001).

**Figure 3:**
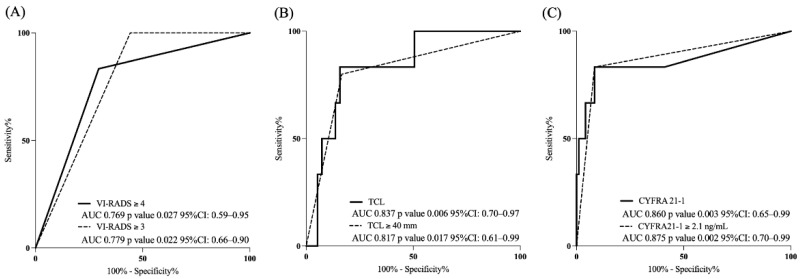
ROC curve analysis of VI-RADS score, TCL, and CYFRA 21-1 levels for predicting 1-year overall survival. (**A**) VI-RADS score; (**B**) TCL; (**C**) CYFRA 21-1 levels. Abbreviations: VI-RADS, Vesical Imaging-Reporting and Data System; AUC, area under the curve; CI, confidence interval; TCL, tumor contact length; CYFRA 21-1, cytokeratin 19 fragment.

ROC analysis showed that a VI-RADS score of 3 had a high predictive ability for 1-year survival (AUC = 0.779). TCL also demonstrated a predictive value for 1-year OS (AUC = 0.837 as a continuous variable). Based on the ROC curve, the optimal cut-off value for TCL was determined to be 40.0 mm using Youden’s index (value = 0.675), resulting in an AUC of 0.817. CYFRA 21-1 levels showed strong predictive ability for the 1-year OS (AUC = 0.860 as a continuous variable). The optimal cut-off value for CYFRA 21-1 was determined to be 2.1 ng/mL using Youden’s index (value = 0.749), yielding an AUC of 0.875 (Figure 3).

According to the composite scoring system that incorporated VI-RADS, TCL, and CYFRA 21-1, patients were stratified into three risk groups: low-risk (0–1 point; n = 81), intermediate-risk (2 points; n = 12), and high-risk (3 points; n = 8). Assuming the observed survival rates in each group (0.92 for the non–high-risk group and 0.25 for the high-risk group), a two-sided α of 0.05, and a total sample size of 101 (93 in the non–high-risk group and 8 in the high-risk group), the calculated power was >0.999. This indicates that the present study had sufficient statistical power to detect the observed survival difference despite the small number of high-risk cases. Table 1 shows the distribution of clinicopathological characteristics across the risk groups. The median age of the study population was 72 years (IQR, 65–81 years). The mean serum CYFRA 21-1 level was 1.64 ng/mL (IQR, 1.0–1.5). Pathological evaluation revealed that 70 patients (69.3%) had NMIBC and 31 (30.7%) had MIBC. High-grade tumors were identified in 68 patients (67.3%). With respect to the imaging findings, organ metastases were present in four patients (4.0%), and lymph node metastases were detected in five (5.0%). Sixteen patients received systemic chemotherapy, and 13 underwent radical cystectomy. When stratified by risk score, patients in the high-risk group had the longest TCL (mean, 68.3 mm; IQR, 48.0–85.7 mm) and the highest CYFRA 21-1 levels (mean, 4.35 ng/mL; IQR, 2.4–5.0). This group also had the highest proportion of MIBC (87.5%), high-grade tumors (100%), and organ (12.5%) and lymph node (12.5%) metastases. In contrast, the low-risk group predominantly consisted of NMIBC cases (82.7%) and showed lower CYFRA 21-1 levels (mean, 1.20 ng/mL; IQR, 1.0–1.3) and shorter TCL values (mean, 13.1 mm; IQR, 7.2–17.2).

**Figure 4:**
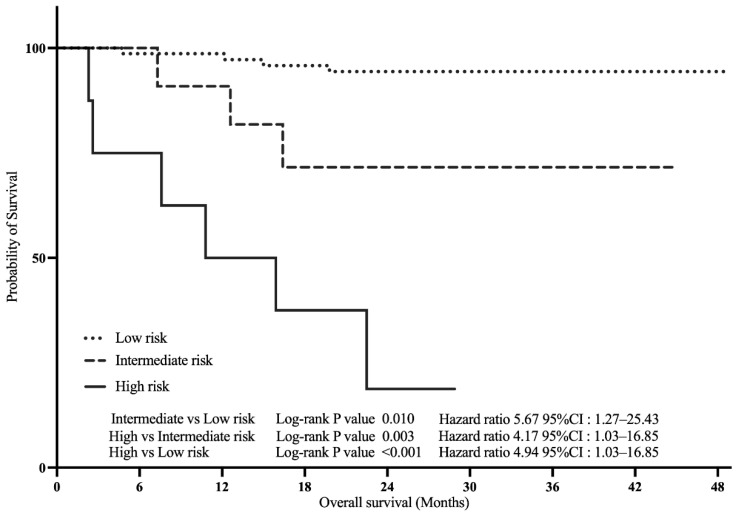
Kaplan–Meier curves for overall survival according to the composite risk classification. Patients were stratified into three groups based on a composite score: one point each for TCL ≥ 40 mm, VI-RADS ≥ 3, and CYFRA 21-1 ≥ 2.1 ng/mL (total score 0–3). Risk groups were defined as low (0–1 points), intermediate (2 points), and high (3 points). Abbreviations: TCL, tumor contact length; VI-RADS, Vesical Imaging-Reporting and Data System; CYFRA 21-1, cytokeratin 19 fragment.

Kaplan–Meier analysis revealed significant differences in OS among the three risk groups (log-rank test, *p* < 0.001; Figure 4). In the Cox proportional hazards regression, with the low-risk group as the reference, the intermediate-risk group showed a significantly higher risk of death (HR, 5.67; 95% CI, 1.27–25.43; *p* = 0.010), and the high-risk group was also associated with an increased risk (HR, 4.94; 95% CI, 1.03–16.85; *p* < 0.001). Furthermore, the high-risk group demonstrated significantly poorer survival compared with the intermediate-risk group (HR, 4.17; 95% CI, 1.03–16.85; *p* = 0.003).

Univariate Cox regression analysis revealed that MIBC, lymph node or organ metastasis, organ metastasis, VI-RADS score ≥ 3, TCL ≥ 40 mm, and CYFRA 21-1 level ≥ 2.1 ng/mL were significantly associated with poor OS (Table 2). Multivariate Cox regression analysis identified CYFRA 21-1 level ≥ 2.1 ng/mL as an independent predictor of a poor OS (HR, 11.80; 95% CI, 2.92–47.73; *p* < 0.001), as shown in Table 2.

**Figure 5:**
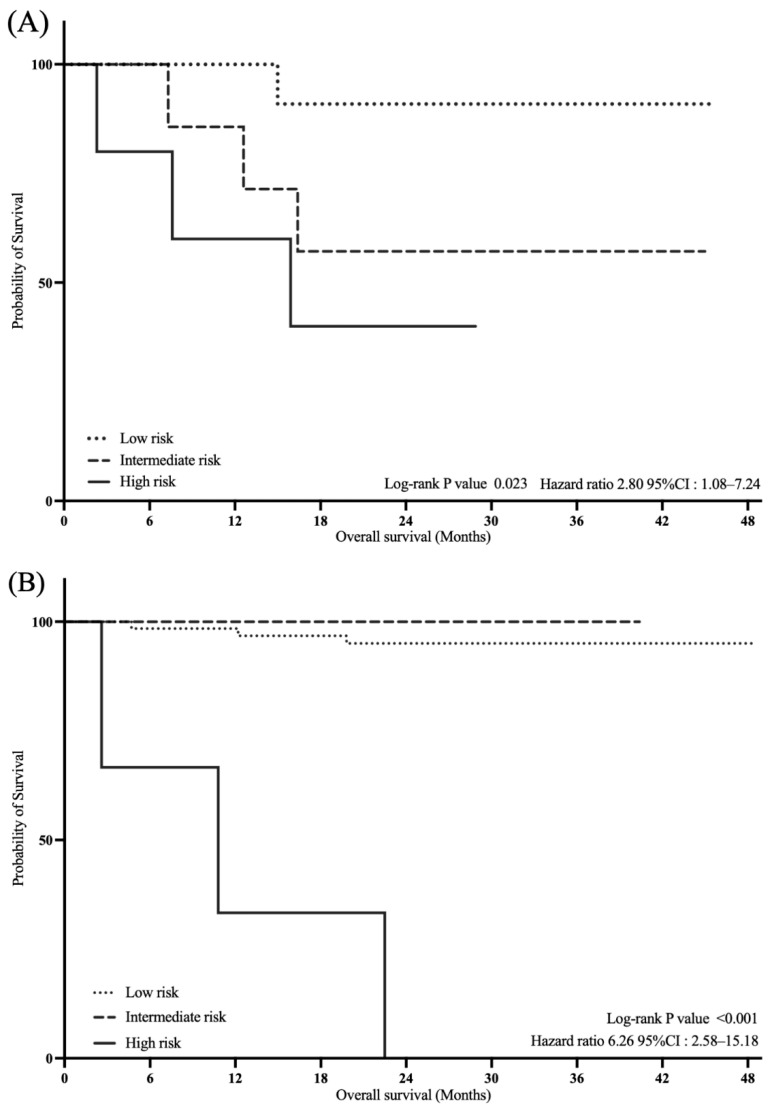
Kaplan–Meier curves for overall survival in subgroup analyses according to treatment status. (**A**) Treated subgroup. (**B**) Untreated subgroup. Abbreviations: CI, confidence interval.

As shown in Figure 5, among the treated patients, the high-risk group had significantly worse OS than the other groups (log-rank, *p* = 0.023; HR, 2.80; 95% CI, 1.08–7.24). Similarly, in the untreated subgroup, the high-risk group showed significantly poorer OS than the other groups (log-rank test, *p* < 0.001; HR, 6.26; 95% CI, 2.58–15.18).

## 4. Discussion

In this study, we explored the prognostic value of integrating the VI-RADS score, serum CYFRA 21-1, and TCL into a composite model for risk stratification in BC. By combining qualitative and quantitative parameters, we developed a risk score that effectively stratified patients into prognostically distinct groups. Unlike conventional nomograms that primarily rely on postoperative pathological findings after radical cystectomy, our study demonstrated that prognostic stratification can be achieved at the peri-TURBT stage using minimally invasive and noninvasive parameters. This represents a novel clinical advantage, as it enables the early identification of high-risk patients before definitive radical treatment, thereby facilitating timely and personalized decision-making. Using this approach, we achieved clear prognostic discrimination, with 3-year OS rates of 95.1%, 75.0%, and 25.0% in the low-, intermediate-, and high-risk groups, respectively. Notably, the high-risk group with all three positive factors showed a strong association with poor prognosis. In our previous study, we demonstrated that combining VI-RADS with serum CYFRA 21-1 allowed noninvasive, multimodal prognostic stratification in patients with BC. Extending this concept, incorporating TCL into a composite risk score provided a more refined stratification into three distinct prognostic groups, thereby supporting earlier intervention at the diagnostic stage of BC and enhancing clinical decision-making.

Several nomograms have been developed to predict BC prognosis. Shariat et al. constructed a model predicting oncological outcomes based on pathological findings after radical cystectomy, including local pathology, lymph node status, and tumor grade [14]. However, within the clinical pathway of BC—which typically includes preoperative MRI, TURBT, and subsequent radical cystectomy or systemic chemotherapy—such nomograms are applicable only after cystectomy. This limits their utility for early clinical intervention. Additionally, current risk stratification tools such as the EORTC and CUETO models were developed before the widespread adoption of multiparametric MRI and modern biomarker research. A recent critical assessment by the EAU Non-Muscle-Invasive Bladder Cancer Guidelines Panel concluded that these traditional models frequently overestimate recurrence and progression risks and no longer reflect contemporary clinical practice or treatment standards [15]. Recently, Yu et al. developed an MRI-based nomogram for the preoperative detection of muscle invasion in VI-RADS 3 lesions, incorporating quantitative MRI-derived features such as TCL [16]. Their findings further underscore the growing importance of quantitative imaging biomarkers in contemporary predictive modeling. Building on these advances, our model enables prognostic prediction at the perioperative TURBT stage by integrating TCL in addition to previously reported factors. In the overall and treated cohorts, this composite model allowed for effective stratification into three prognostic groups. Furthermore, in the untreated subgroup, the high-risk group showed a markedly worse prognosis than the low- and intermediate-risk groups. This approach may be particularly valuable for determining the need for post-TURBT treatment and for selecting candidates for neoadjuvant chemotherapy or bladder-sparing strategies, thereby supporting timely and personalized clinical decision-making in BC management.

The VI-RADS was introduced in 2018 by Panebianco et al. as a standardized reporting system for assessing muscle invasion in BC [1]. Its high diagnostic accuracy for MIBC has been confirmed in meta-analyses and has since been widely adopted [2]. Previous studies have reported that higher VI-RADS scores are associated with poorer prognosis, with BC, with significantly reduced OS observed in patients with VI-RADS scores ≥ 3 (HR, 3.517; *p* = 0.003) [17]. Consistent with these findings, our univariate analysis showed that VI-RADS ≥ 3 predicted poor prognosis (HR, 6.51; *p* = 0.015). Similarly, pathological findings of MIBC based on TURBT were significant in univariate Cox regression analysis (HR, 6.05; *p* = 0.003). Since MIBC itself represents an adverse prognostic factor and VI-RADS functions as a predictive tool for muscle invasion, it is reasonable that higher VI-RADS scores correlate with poorer outcomes. Collectively, these findings suggest that VI-RADS may serve not only as a predictor of muscle invasion, but also as a valuable prognostic indicator in BC.

In this study, we adopted TCL as a novel factor to extend the functionality of VI-RADS for prognostic prediction. Empirically, it has been recognized that broad-based tumors in BC are associated with an increased risk of MIBC. Wang et al. reported that bladder tumors lacking a stalk on MRI were more likely to represent MIBC [18]. With respect to the quantification of TCL and its correlation with muscle invasion, Ozden et al. demonstrated that tumors with a TCL of ≥41.5 mm, as assessed by ultrasonography, were significantly associated with MIBC [8]. Akcay et al. proposed a cut-off value of 19.5 mm for diagnosing muscle invasion and reported a very high diagnostic accuracy with an AUC of 0.98 [11]. Similarly, Selvaraju et al. showed that TCL was effective, with a threshold of 30 ± 3 mm (AUC, 0.90–0.92) [9]. TCL is increasingly being incorporated into diagnostic assessments as a quantitative factor complementing VI-RADS. Ahn et al. suggested that TCL ≥ 30 mm within VI-RADS 2–3 cases could allow further risk stratification for MIBC prediction [10]. Additionally, Li et al. demonstrated that combining tumor contact length with diffusion kurtosis imaging improved the detection of muscle invasiveness in bladder cancer, further supporting the value of TCL as a quantitative imaging biomarker [19]. In the present study, we analyzed TCL as a new prognostic parameter for BC. As reflected in the EORTC risk tables developed by Sylvester et al., tumor size is widely recognized as a risk factor for recurrence and progression [12]. TCL reflects tumor size, and when representing the maximal tumor diameter, can provide a quantitative description of tumor morphology. This finding supports the hypothesis that TCL may serve as a prognostic factor; therefore, it was included in our analysis. In our model, TCL ≥ 40 mm was identified as a poor prognostic factor in univariate analysis (HR, 8.36; *p* < 0.001). Furthermore, by incorporating TCL into our previously established prognostic model based on VI-RADS and CYFRA 21-1, we achieved a more refined stratification of patients with BC.

In our model, CYFRA 21-1 was a strong prognostic factor. Cytokeratin is overexpressed in BC [20]. Serum CYFRA 21-1 has been recognized as a useful diagnostic marker for BC [5]. Furthermore, Daniel et al. reported that CYFRA 21-1 levels were elevated in MIBC [21], and Andreadis et al. demonstrated significantly higher CYFRA 21-1 levels in patients with metastatic BC [22]. Higher CYFRA 21-1 levels were also significantly associated with higher tumor stage and grade [23]. A meta-analysis further confirmed a significant association between elevated CYFRA 21-1 and the presence of bladder cancer [24], supporting its clinical relevance. In our study, the ROC analysis for predicting 1-year survival showed a strong correlation (AUC = 0.86; *p* < 0.001). In addition, multivariate analysis revealed that CYFRA 21-1 ≥ 2.1 ng/mL was an independent prognostic factor, indicating high risk with an HR of 11.80. According to Andreadis et al., 66% of metastatic cases showed elevated CYFRA 21-1 levels [19]. In our previous study, serum CYFRA 21-1 levels were significantly correlated with lymph node and visceral metastases [7]. By adding TCL to VI-RADS, we improved the accuracy of local diagnosis. Furthermore, incorporating CYFRA 21-1, which correlates positively with metastatic disease, as a complementary factor enabled us to build a three-factor model that more precisely stratified patients with a poor prognosis.

This study differs from existing nomograms, which were constructed based on clinical factors and pathological findings from cystectomy specimens. This is because it is a prognostic model based on qualitative and quantitative imaging findings combined with serum biomarkers. Adding the quantified morphological feature of TCL to the qualitative imaging parameter of the VI-RADS score represents an improvement over our previous research. The approach of incorporating image-derived features into predictive models is supported by studies such as those by Huang et al. [25], where a deep learning (DL) model combining MRI-derived radiomics features and clinical factors accurately predicted the recurrence risk in NMIBC, and a prognostic model integrating CT-derived radiomics features and clinical data predicted the outcomes in patients with MIBC [26]. Furthermore, Yang et al. recently reported that a radiomics–clinical nomogram based on multi-sequence MRI improved recurrence-risk prediction compared with clinical models alone in patients with bladder cancer, highlighting the growing importance of MRI-derived quantitative features in prognostic assessment [27]. Compared with these studies, the advantage of our method is that it provides a relatively simple quantitative measurement, whereas radiomics and DL research require significant time and cost for annotation and analysis. This model does not require a specialized image analysis infrastructure and can be feasibly implemented by urologists in routine clinical practice. Furthermore, potential issues remain in which humans cannot visualize or explain the diagnosis. In the future, we aim to develop radiomics and DL systems that incorporate morphological features such as VI-RADS and TCL, both of which have been identified as adverse prognostic indicators. In particular, radiomics has been reported in several studies to contribute to the identification of adverse prognostic factors in BC [28,29,30]. By integrating these systems into imaging diagnostics, we hope to further enhance risk stratification and evaluate their potential contribution to personalized treatment strategies.

This study had some limitations. First, this was a retrospective, single-center study, which may limit the generalizability of the results. Second, the proposed model was not externally validated; prospective studies are required to confirm its utility. External validation should be performed before clinical implementation. Third, although the MRI protocol followed the VI-RADS guidelines, variations in imaging conditions may have affected reproducibility. Fourth, CYFRA 21-1 levels may vary depending on the timing of blood collection. In this study, measurements were performed postoperatively; however, preoperative data were unavailable, and variations in the timing of blood sampling may have introduced bias related to tumor burden. Moreover, CYFRA 21-1 is not reimbursed by insurance in Japan; therefore, it was not measured in all patients. Fifth, the number of high-risk patients was relatively small, which may have limited the statistical robustness of subgroup analyses. However, this distribution reflects the actual prevalence of high-risk disease in daily clinical practice. Despite these limitations, this study provides valuable insights into the potential of combining imaging and serum biomarkers for BC risk stratification.

## 5. Conclusions

This study extends our previous work by incorporating TCL into a multimodal prognostic model based on VI-RADS and CYFRA 21-1. We demonstrated that integrating three complementary factors—qualitative (VI-RADS), quantitative morphological (TCL), and serological (CYFRA 21-1)—enables the effective stratification of patients with BC into prognostically distinct groups. In particular, the high-risk group, defined by positivity for all three factors, showed markedly poorer survival outcomes, highlighting the clinical relevance of the composite risk score. Unlike previous nomograms that relied on postoperative pathological findings, our model is based on peri-TURBT, a minimally invasive assessment that highlights its potential for early risk stratification and treatment decision-making. Future prospective multicenter studies are warranted to validate the generalizability of this model and further refine its role in guiding personalized treatment strategies for BC.

## Figures and Tables

**Table 1:** Patient characteristics according to the composite risk classification based on VI-RADS, TCL, and CYFRA 21-1.

	Total *n* = 101	Low Risk (Score ≤ 1) *n* = 81	Intermediate Risk (Score 2) *n* = 12	High Risk (Score 3) *n* = 8	*p*-Value
Baseline Characteristics					
Age					0.875
Median, [IQR]	73.0 [64.5–81.0]	73.0 [64.5–81.0]	74.5 [58.5–82.5]	72.5 [69.0–82.5]	
Mean ± SD (years)	72.1 ± 10.3	71.9 ± 10.1	72.3 ± 12.3	73.9 ± 9.6	
Sex					0.942
Men, *n*, (%)	84 (83.2)	67 (82.7)	10 (83.3)	7 (87.5)	
Women, *n*, (%)	17 (16.8)	14 (17.3)	2 (16.7)	1 (12.5)	
VI-RADS					<0.001
Score 1, *n*, (%)	16 (15.8)	16 (19.8)	0 (0)	0 (0)	
Score 2, *n*, (%)	37 (36.6)	37 (45.7)	0 (0)	0 (0)	
Score 3, *n*, (%)	15 (14.9)	15 (18.5)	0 (0)	0 (0)	
Score 4, *n*, (%)	5 (5.0)	2 (2.5)	2 (16.7)	1 (12.5)	
Score 5, *n*, (%)	28 (27.7)	11 (13.6)	10 (83.3)	7 (87.5)	
TCL					<0.001
Median, [IQR]	14.32, [8.53–27.11]	12.04, [7.20–17.16]	53.72, [44.73–74.54]	68.94, [48.00–85.74]	
Mean ± SD (mm)	23.02 ± 22.61	13.14 ± 7.52	59.60 ± 18.08	68.28 ± 19.47	
CYFRA 21-1					<0.001
Median, [IQR]	1.0, [1.0–1.5]	1.0, [1.0–1.3]	1.35, [1.0–1.7]	3.10, [2.4–5.0]	
Mean ± SD (ng/mL)	1.64 ± 0.21	1.20 ± 0.38	2.81 ± 5.11	4.35 ± 3.27	
Pathological Findings					
T-stage					<0.001
NMIBC, *n*, (%)	70 (69.3)	67 (82.7)	2 (16.7)	1 (12.5)	
MIBC, *n*, (%)	31 (30.7)	14 (17.3)	10 (83.3)	7 (87.5)	
Tumor grade					0.012
High, *n*, (%)	68 (67.3)	49 (60.5)	11 (91.7)	8 (100)	
Low, *n*, (%)	33 (32.7)	32 (39.5)	1 (8.3)	0 (0)	
Carcinoma in situ					
Presence, *n*, (%)	10 (9.9)	10 (100)	0 (0)	0 (0)	
Absence, *n*, (%)	91 (90.1)	0 (0)	0 (0)	0 (0)	0.254
Imaging Findings Metastasis					
Organ, *n*, (%)	4 (4.0)	1 (1.2)	2 (16.7)	1 (12.5)	0.016
Lymph node, *n*, (%)	5 (5.0)	1 (1.2)	3 (25.0)	1 (12.5)	0.001
Treatment Variables					
Systemic chemotherapy					<0.001
GC, *n*, (%)	16 (15.8)	9 (11.1)	6 (50.0)	1 (12.5)	
GCb, *n*, (%)	3 (3.0)	0 (0)	1 (8.3)	2 (25.0)	
Pembrolizumab, *n*, (%)	4 (4.0)	1 (1.2)	2 (16.7)	1 (12.5)	
Avelmab, *n*, (%)	3 (3.0)	1 (1.2)	2 (16.7)	0 (0)	
Enfortumab Vedotin, *n*, (%)	1 (0.9)	0 (0)	1 (8.3)	0 (0)	
Cystectomy, *n*, (%)	13 (12.9)	7 (8.6)	2 (16.7)	4 (50.0)	0.004

Abbreviations: IQR, interquartile range; SD, standard deviation; VI-RADS, Vesical Imaging-Reporting and Data System; TCL, tumor contact length; CYFRA 21-1, cytokeratin 19 fragment; NMIBC, non–muscle-invasive bladder cancer; MIBC, muscle-invasive bladder cancer; GC, gemcitabine and cisplatin; GCb, gemcitabine and carboplatin.

**Table 2:** Cox regression analysis of prognostic factors for overall survival.

	Univariate HR (95% CI)	*p*-Value	Multivariate HR (95% CI)	*p*-Value
Age	1.00 (0.95–1.06)	0.928	–	–
MIBC	6.05 (1.86–19.69)	0.003	0.98 (0.15–6.21)	0.978
High grade	38.68 (0.31–4829.82)	0.138	–	–
Lymph node or organ metastasis	4.25 (1.17–15.48)	0.028	2.83 (0.63–12.73)	0.175
VI-RADS ≥ 3	6.51 (1.44–29.38)	0.015	4.18 (0.54–32.15)	0.170
TCL ≥ 40 mm	8.36 (2.72–25.66)	<0.001	1.26 (0.24–6.76)	0.786
CYFRA 21-1 ≥ 2.1 ng/mL	14.02 (4.56–43.12)	<0.001	11.80 (2.92–47.73)	<0.001
Systemic chemotherapy	2.49 (0.81–7.62)	0.110	–	–
Cystectomy	3.03 (0.93–9.83)	0.066	–	–

Abbreviations: MIBC, muscle-invasive bladder cancer; VI-RADS, Vesical Imaging-Reporting and Data System; TCL, tumor contact length; CYFRA 21-1, cytokeratin 19 fragment.

## Data Availability

The data supporting the findings of this study are available from the corresponding author upon reasonable request. The data are not publicly available due to patient privacy restrictions.

## Abbreviations

The following abbreviations are used in this manuscript:

VI-RADS	The Vesical Imaging-Reporting and Data System
BC	Bladder cancer
MRI	Magnetic resonance imaging
MIBC	Muscle-invasive bladder cancer
NMIBC	Non-muscle-invasive bladder cancer
CYFRA 21-1	Cytokeratin 19 fragment
TCL	Tumor contact length
TURBT	Transurethral resection of bladder tumor
IRB	Institutional Review Board
T2WI	T2-weighted imaging
DWI	Diffusion-weighted imaging
DCE	Dynamic contrast-enhanced
ROC	Receiver operating characteristic
OS	Overall survival
HR	Hazard ratio
CI	Confidence interval
ICC	Intraclass correlation coefficient
AUC	Area under the curve
IQR	Interquartile range
DL	Deep learning

## References

[1] Panebianco V., Narumi Y., Altun E., Bochner B.H., Efstathiou J.A., Hafeez S., Huddart R., Kennish S., Lerner S., Montironi R. (2018). Multiparametric Magnetic Resonance Imaging for Bladder Cancer: Development of VI-RADS (Vesical Imaging-Reporting and Data System). Eur. Urol..

[2] Del Giudice F., Flammia R.S., Pecoraro M., Moschini M., D’Andrea D., Messina E., Pisciotti L.M., De Berardinis E., Sciarra A., Panebianco V. (2022). The Accuracy of Vesical Imaging-Reporting and Data System (VI-RADS): An Updated Comprehensive Multi-Institutional, Multi-Readers Systematic Review and Meta-Analysis from Diagnostic Evidence into Future Clinical Recommendations. World J. Urol..

[3] Doshi C., Zahir M., Dadabhoy A., Escobar D., Xia L., Daneshmand S. (2025). Serum Tumor Markers for Muscle-Invasive Bladder Cancer in Clinical Practice: A Narrative Review. Cancers.

[4] Sundström B.E., Stigbrand T.I. (1994). Cytokeratins and Tissue Polypeptide Antigen. Int. J. Biol. Markers.

[5] Huang Y.L., Chen J., Yan W., Zang D., Qin Q., Deng A.M. (2015). Diagnostic Accuracy of Cytokeratin-19 Fragment (CYFRA 21-1) for Bladder Cancer: A Systematic Review and Meta-Analysis. Tumour Biol..

[6] Senga Y., Kimura G., Hattori T., Yoshida K. (1996). Clinical Evaluation of Soluble Cytokeratin 19 Fragments (CYFRA 21-1) in Serum and Urine of Patients with Bladder Cancer. Urology.

[7] Ikuma S., Akatsuka J., Kaneko G., Takeda H., Endo Y., Kimura G., Kondo Y. (2025). Prognostic Utility of Combining VI-RADS Scores and CYFRA 21-1 Levels in Bladder Cancer: A Retrospective Single-Center Study. Curr. Oncol..

[8] Ozden E., Turgut A.T., Yesil M., Gögüs C., Gögüs O. (2007). A New Parameter for Staging Bladder Carcinoma: Ultrasonographic Contact Length and Height-to-Length Ratio. J. Ultrasound Med..

[9] Selvaraju A., Patbamniya N.K., Kumar M., Seth A., Kaushal S., Das C.J. (2025). Preoperative Prediction of Muscle Invasion in Bladder Cancer in the Indian Population Using the Vesical Imaging-Reporting and Data System (VI-RADS) Score and Individual Multiparametric Magnetic Resonance Imaging (MRI) Characteristics. Clin. Radiol..

[10] Ahn H., Kim T.M., Hwang S.I., Lee H.J., Choe G., Hong S.K., Byun S.-S., Oh J.J. (2023). Tumor Contact Length with Bladder Wall Provides Effective Risk Stratification for Lesions with a VIRADS Score of 2–3. Eur. Radiol..

[11] Akcay A., Yagci A.B., Celen S., Ozlulerden Y., Turk N.S., Ufuk F. (2021). VI-RADS Score and Tumor Contact Length in MRI: A Potential Method for the Detection of Muscle Invasion in Bladder Cancer. Clin. Imaging.

[12] Sylvester R.J., van der Meijden A.P.M., Oosterlinck W., Witjes J.A., Bouffioux C., Denis L., Newling D.W.W., Kurth K. (2006). Predicting Recurrence and Progression in Individual Patients with Stage Ta T1 Bladder Cancer Using EORTC Risk Tables: A Combined Analysis of 2596 Patients from Seven EORTC Trials. Eur. Urol..

[13] Noh T.I., Shim J.S., Kang S.G., Sung D.J., Cheon J., Sim K.C., Kang S.H. (2022). Comparison between Biparametric and Multiparametric MRI in Predicting Muscle Invasion by Bladder Cancer Based on the VI-RADS. Sci. Rep..

[14] Shariat S.F., Karakiewicz P.I., Palapattu G.S., Lotan Y., Rogers C.G., Amiel G.E., Vazina A., Gupta A., Bastian P.J., Sagalowsky A.I. (2006). Outcomes of Radical Cystectomy for Transitional Cell Carcinoma of the Bladder: A Contemporary Series from the Bladder Cancer Research Consortium. J. Urol..

[15] Soukup V., Čapoun O., Cohen D., Hernández V., Burger M., Compérat E., Gontero P., Lam T., Mostafid A.H., Palou J. (2020). Risk Stratification Tools and Prognostic Models in Non-muscle-invasive Bladder Cancer: A Critical Assessment from the European Association of Urology Non-muscle-invasive Bladder Cancer Guidelines Panel. Eur. Urol. Focus.

[16] Yu R., Cai L., Cao Q., Liu P., Gong Y., Li K., Wu Q., Zhang Y., Li P., Yang X. (2023). Development and Validation of an MRI-Based Nomogram for Preoperative Detection of Muscle Invasion in VI-RADS 3. J. Magn. Reson. Imaging.

[17] Zhuang J., Cai L., Sun H., Wu Q., Li K., Yu R., Cao Q., Li P., Yang X., Lu Q. (2023). Vesical Imaging Reporting and Data System (VI-RADS) Could Predict the Survival of Bladder-Cancer Patients Who Received Radical Cystectomy. Sci. Rep..

[18] Wang H.J., Pui M.H., Guan J., Li S.R., Lin J.H., Pan B., Guo Y. (2016). Comparison of Early Submucosal Enhancement and Tumor Stalk in Staging Bladder Urothelial Carcinoma. AJR Am. J. Roentgenol..

[19] Li Q., Cao B., Liu K., Sun H., Ding Y., Yan C., Wu P.-Y., Dai C., Rao S., Zeng M. (2022). Detecting the Muscle Invasiveness of Bladder Cancer: An Application of Diffusion Kurtosis Imaging and Tumor Contact Length. Eur. J. Radiol..

[20] Mady E.A. (2001). Cytokeratins as Serum Markers in Egyptian Bladder Cancer: A Comparison of CYFRA 21-1, TPA and TPS. Int. J. Biol. Markers.

[21] Fatela-Cantillo D., Fernández-Suárez A., Menéndez V., Galán J.A., Filella X. (2005). Low Utility of CYFRA 21-1 Serum Levels for Diagnosis and Follow-Up in Bladder Cancer Patients. J. Clin. Lab. Anal..

[22] Andreadis C., Touloupidis S., Galaktidou G., Kortsaris A.H., Boutis A., Mouratidou D. (2005). Serum CYFRA 21-1 in Patients with Invasive Bladder Cancer and Its Relevance as a Tumor Marker during Chemotherapy. J. Urol..

[23] Washino S., Hirai M., Matsuzaki A., Kobayashi Y. (2011). Clinical Usefulness of CEA, CA19-9, and CYFRA 21-1 as Tumor Markers for Urothelial Bladder Carcinoma. Urol. Int..

[24] Kuang L.I., Song W.J., Qing H.M., Yan S., Song F.L. (2015). CYFRA21-1 Levels Could Be a Biomarker for Bladder Cancer: A Meta-Analysis. Genet. Mol. Res..

[25] Huang H., Huang Y., Kaggie J.D., Cai Q., Yang P., Wei J., Wang L., Guo Y., Lu H., Wang H. (2025). Multiparametric MRI-Based Deep Learning Radiomics Model for Assessing 5-Year Recurrence Risk in Non-Muscle Invasive Bladder Cancer. J. Magn. Reson. Imaging.

[26] Wang B., Gong Z., Su P., Zhen G., Zeng T., Ye Y. (2025). Multi-Machine Learning Model Based on Radiomics Features to Predict Prognosis of Muscle-Invasive Bladder Cancer. BMC Cancer.

[27] Yang G., Bai J., Hao M., Zhang L., Fan Z., Wang X. (2024). Enhancing Recurrence Risk Prediction for Bladder Cancer Using Multi-Sequence MRI Radiomics. Insights Imaging.

[28] Cao T., Li N., Guo C., Zhang H., Chen L., Wu K., Liang L., Wang X., Shen W. (2025). CT-based Radiomics of Intratumoral and Peritumoral Regions to Predict the Recurrence Risk in Patients with Non-muscle-invasive Bladder Cancer within Two Years after TURBT. Curr. Med. Imaging..

[29] Park K.J., Lee J.-L., Yoon S.-K., Heo C., Park B.W., Kim J.K. (2020). Radiomics-Based Prediction Model for Outcomes of PD-1/PD-L1 Immunotherapy in Metastatic Urothelial Carcinoma. Eur. Radiol..

[30] Wang W., Wang K., Qiu J., Li W., Wang X., Zhang Y., Wang X., Wu J. (2023). MRI-Based Radiomics Analysis of Bladder Cancer: Prediction of Pathological Grade and Histological Variant. Clin. Radiol..

